# Our Semi-centennial Anniversary

**Published:** 1895-07

**Authors:** 


BUFFALO MEDICAL AND SURGICAL JOURNAL

A MONTHLY REVIEW OF MEDICINE AND SURGERY.

*EDITORS:*

THOMAS LOTHROP, M. D. - - WM. WARREN POTTER, M. D.

All communications, whether of a literary or business nature, should be addressed to the managing editor: 284 Franklin Street, Buffalo, N. Y.

Vol. XXXIV. JULY, 1895. No. 12.

### OUR SEMI-CENTENNIAL ANNIVERSARY.

With this issue the Buffalo Medical Journal closes the first half century of its career. Were we disposed to avail ourselves of this opportunity we might furnish some reminiscences of a pleasant if not interesting character. But as we propose in our next issue to give a historical résumé of journalism and medicine in Buffalo during the last fifty years, which will be profusely illustrated, we will reserve such comments for that occasion.

It is our object at this time, however, to call particular attention to the fact that our next volume, beginning August, 1895, will contain 960 pages of reading matter, or eighty pages in each monthly issue. It will be printed on handsome book-paper of uniform quality from cover to cover, thus giving our advertisers equal advantage with our readers in respect to quality of paper.

The August number will be as beautiful a specimen of the medical magazine art as can be produced. It will be essentially a Buffalo number, as all its contributions will be from the pens of members of the profession residing in Buffalo, many of which will be illustrated.

Among those who have promised contributions for the August number we may mention Herman Mynter, Roswell Park, Matthew D. Mann, Alvin A. Hubbell, Julius Pohlman, Edward Clark, William C. Krauss, Charles G. Stockton, C. M. Daniels, John A. Miller, John Parmenter, Irving M. Snow, Bernard Bartow, Henry D. Ingraham, F. Whitehill Hinkel, James W. Putnam, Carlton C. Frederick, Ernest Wende, Charles Cary, F. S. Crego and Henry R. Hopkins. Many of these papers will be artistically illustrated.

In addition it will contain a historical résumé of medical affairs in Buffalo during the last fifty years. Besides an account of medical journals this will deal with hospitals, medical colleges, medical societies and other subjects of professional interest. This article also will be elaborately illustrated.

A large special edition of the August number will be printed, which will be sold at the low price of 25 cents a copy. All who are not subscribers (or if they desire extra copies) should place their orders at once to enable us to determine the number to print. Every physician educated in Buffalo will be interested in this special number as well as all who are residing in Western New York. Lawyers, too, and others not of the medical profession will find much of interest in its columns. Let us have your orders accompanied by the money—25 cents a copy—at once.

